# Effects of RNA interference-mediated gene silencing of JMJD2A on human breast cancer cell line MDA-MB-231 in vitro

**DOI:** 10.1186/1756-9966-30-90

**Published:** 2011-10-03

**Authors:** Bei-Xu Li, Ming-Chang Zhang, Cheng-Liang Luo, Peng Yang, Hui Li, Hong-Mei Xu, Hong-Fei Xu, Yi-Wen Shen, Ai-Min Xue, Zi-Qin Zhao

**Affiliations:** 1 Department of Forensic Medicine, Shanghai Medical College, Fudan University, Shanghai 200032, PR China

**Keywords:** JMJD2A, transfection, proliferation, invasion, migration

## Abstract

Previous data demonstrate that JMJD2A is a cancer-associated gene and may be involved in human breast cancer by demethylation of H3K9me3. The aim of this study was to investigate depressive effects on JMJD2A by transfection with JMJD2A-sepcific siRNA in human breast cancer cell line MDA-MB-231 and effects on cell proliferation, invasion and migration. JMJD2A-specific siRNA was chemically synthesised and transfected into human breast cancer cell line MDA-MB-231. Expression levels of JMJD2A were detected by quantitative real-time PCR and Western blot analysis. Cells proliferation was evaluated by using flow cytometric anlysis and MTT assay. The abilities of invasion and migration were evaluated by cell migration and invasion assay with Boyden chambers. The results showed that the transfection was successful and expression levels of JMJD2A mRNA and protein in siRNA group were both down-regulated. By MTT assay, the mean actual absorbance in siRNA group was significantly lower than that in blank control group (P < 0.05) and negative control group (P < 0.05). In addition, the percentage of cells in G0/G1 phase in siRNA group was significantly more than that in blank control group (P < 0.05) and negative control group (P < 0.05). Furthermore, by cell invasion and migration assay, the decreased number of migrated cells in siRNA group was observed (P < 0.05). These data imply that silencing JMJD2A gene could result in cell cycle change and proliferation inhibition, and lead to suppress tumor cell invasion and migration. It provides a new perspective in understanding the pleiotropic functions of JMJD2A and its contribution to human breast cancer.

## Background

Human breast cancer is one of the most frequent malignant tumors with the incidence rate increasing year by year. Based on the GLOBOCAN 2008 estimates, breast cancer is the most frequently diagnosed cancer and the leading cause of cancer death among females, accounting for 23% of the total cancer cases and 14% of the cancer deaths [1]. The prognosis of the patients with advanced stage breast cancer is poor, because of the progression and metastasis of the disease, even surgical removal, chemotherapy and endocrine therapy were employed for most cases. Prevention and treatment of breast cancer require a better understanding of the molecular mechanisms underlying the progression of breast cancer.

Gene therapies for tumor were focused on in recent years, including gene replacement, antisense nucleic acid technique, cytokine gene therapy and RNA interference (RNAi) technique. RNAi is a post-transcriptional regulation and provides a rapid means of depleting mRNAs by introducing double-stranded RNA homologous to a particular message leading to its sequence-specific degradation. It is simple, specific and effective to use small interfering RNA (siRNA) to silence target gene [2].

Jumonji Domain Containing 2A (JMJD2A, also known as JHDM3 or KDM4A) was identified and characterized in 2004 [3]. JMJD2A belongs to the JmjC domain-containing family JMJD2 proteins, which are lysine trimethyl-specific histone demethylases catalyzing the demethylation of trimethylated H3K9 (H3K9me3) and H3K36 (H3K36me3) [4-6]. JMJD2 family genes are cancer-associated genes [3]. JMJD2A is widely expressed in human tissues and cell lines, and high endogenous expression of JMJD2A mRNA was found in several cell types, including human T-cell lymphotropic virus 1-infected cell lines, the HT1376 bladder carcinoma cell line, the U2OS osteosarcoma cell line and the prostate cancer cell line [7,8]. However, there are rare literatures focusing on the relationship between JMJD2A and breast cancer.

In this study, JMJD2A-specific siRNA was chemically synthesised and transfected into human breast cancer cell line MDA-MB-231. The levels on JMJD2A mRNA and its protein expression, and biological characteristics of MDA-MB-231 cells including proliferation, migration and invasion were investigated.

## Materials and methods

### JMJD2A siRNA synthesis

JMJD2A siRNA was chemically synthesised by Qiagen Technology Co. Ltd (Shanghai, China). siRNA was diluted to 20 μmol/L with free-RNase water. siRNA duplexes were synthesised as follows: Sense sequence: 5'-GAGUUAUCAACUCAAGAUA-3', Antisense sequence: 5'-UAUCUUGAGUUGAUAACUC-3'.

### Cell transfection

Human breast cancer cell line MDA-MB-231 in this research was preserved in our laboratory. At 24 h before transfection, MDA-MB-231 cells in logarithmic growth phase were seeded into 6-well plates, at a density of 5 × 10^5 ^cells per well and incubated in RPMI 1640 medium (GIBCO, Invitrogen, USA) containing 10% FBS (GIBCO, Invitrogen, USA). RPMI 1640 medium containing 10% FBS was replaced by serum-free Opti-MEM (GIBCO, Invitrogen, USA) at 8 h later. HiPerFect Transfection Reagent and Negative control siRNA were purchased from Qiagen Technology Co. Ltd (Shanghai, China). Transfection compounds were prepared in three groups as follows: siRNA group (100 μl Opti-MEM, 6 μl HiPerFect Transfection Reagent and 5 μl JMJD2A siRNA), negative control group (100 μl Opti-MEM, 6 μl HiPerFect Transfection Reagent and 5 μl negative control siRNA) and blank control group (100 μl Opti-MEM). Transfection compounds were placed at room temperature for 10 minutes and then dropped onto 6-well plates. Bulk volume of the compounds was 2200 μl per well. Both Opti-MEM and transfection compounds were replaced by complete medium at 24 h after transfection. FAM-siRNA was transfected to measure the efficiency of transfection simultaneously according to the manufacturer's instructions.

### Quantitative real-time PCR

Total RNA of three groups was extracted respectively with the RNAiso Reagent kit (TaKaRa, Dalian, China) at 48 h after transfection. cDNA was generated by reverse transcription of 2 μg of total RNA using random primers and PrimeScript RT Master Mix Perfect Real Time (TaKaRa, Dalian, China) in a total reaction volume of 40 μl according to the manufacturer's instructions. The sequences of forward and reverse oligonucleotide primers, specific to JMJD2A and housekeeping genes, were designed using Primer5 software. The primers used are: 5'-TGTGCTGTGCTCCTGTAG -3' and 5'-GTCTCCTTCCTCTCCATCC -3' for JMJD2A; 5'-TGACGCTGGGGCTGGCATTG -3' and 5'-GCTCTTGCTGGGGCTGGTGG -3' for GAPDH. Primers were synthesised by Shanghai Daweike Biotechnology Co. Ltd (Shanghai, China).

Real-time quantitative PCR was performed in an ABI PRISM 7500 Real-Time System. A 10-fold dilution of each cDNA was amplified in a 20-μl volume, using the SYBR Premix Ex TaqTM Perfect Real Time (TaKaRa, Dalian, China), with 0.2 μM final concentrations of each primer. PCR cycle conditions were 95°C for 30 s, and 40 cycles of 95°C for 5 s and 60°C for 34 s. The amplification specificity was evaluated with melting curve analysis. Threshold cycle Ct, which correlates inversely with the target mRNA levels, was calculated using the second derivative maximum algorithm provided by the iCycler software. For JMJD2A, the mRNA levels were normalized to GAPDH mRNA levels [9].

### Western blot

At 72 h after transfection, cells in different treatment groups were homogenized in Western blot analysis buffer containing 10 mM Tris-HCl (pH 7.4), 150 mM NaCl, 1% (v/v) Triton X-100, 1% sodium deoxycholate, 0.1% SDS, 5 mM EDTA, 1 mM PMSF, 0.28 kU/L aprotinin, 50 mg/L leupeptin, 1 mM benzamidine and 7 mg/L pepstain A. The homogenate was then centrifuged at 12, 000 rpm for 10 min at 4°C and the supernatant was retained and preserved at -80°C for later use. Protein concentration was determined using a BCA kit (Pierce). Twenty micrograms of protein from each group were subject to electrophoresis on 10% SDS-PAGE gel using a constant current. Proteins were transferred to nitrocellulose membranes on a semidry electrotransferring unit and incubated with monoclonal rabbit anti-human JMJD2A antibody (Cell Signaling Technology, USA, 1:1000) in Tris-buffered saline containing 0.1% Tween-20 (TBST) and 5% nonfat dry milk overnight at 4°C. After the overnight incubation with the primary antibodies, membranes were washed and incubated with HRP-labelled goat anti-rabbit second antibody (Santa Cruz Biotechnology Inc., USA) in TBST for 2 h. Immunoreactivity was detected with enhanced chemoluminescent autoradiography (ECL kit, Amersham), according to the manufacturer's instructions. The membranes were reprobed with GAPDH (Cell Signaling Technology, USA, 1:1000) after striping. The signal intensity of primary antibody binding was quantitatively analyzed with Sigma Scan Pro 5 and was normalized to a loading control, GAPDH [10].

### Flow cytometric anlysis (FCM)

At 72 h after transfection, cells in different treatment groups were collected with trypsinization, then washed with PBS twice. Cells were fixed in 70% ethanol for 1 h at room temperature. After centrifugation, the cell pellet was resuspended in PBS (pH 7.4), containing 100 μL RNase A (1 mg/mL) and 400 μL propidium iodide (50 μg/mL). The cells were incubated for 30 min at room temperature, and DNA content was determined by flow cytometry using a FACScan flow cytometer at 488 nm and the data were input to computer and analyzed by software Light cycle. The experiment was performed three times in triplicate [11]. Proliferation indexes (PI) was calculated as follows: PI = (S+G2/M)/(G0/G1+S+G2/M)×100%.

### MTT assay

MDA-MB-231 cells were seeded into 96-well plates at a density of 1 × 10^4 ^cells per well and incubated in RPMI 1640 medium containing 10% FBS. RPMI 1640 medium containing 10% FBS was replaced by serum-free Opti-MEM 8 h later. These cells were grouped as indicated above (*cell transfection*). The bulk volume of the transfection compounds was 100 μl per well. Opti-MEM and transfection compounds were replaced by complete medium at 24 h after transfection. After 72 h of incubation, MDA-MB-231 cells were incubated for an additional 4 hours with 20 μl MTT (Sigma Chemical Co., USA, 5 mg/ml). Then the supernatant was removed, and 150 μl DMSO was added. Absorbance at 570 nm (A570) of three groups and DMSO (Sigma Chemical Co., USA) was measured with a microplate reader (Model 550, Bio-Rad, USA) [11]. All experiments were carried out eight times. Actual absorbance = absorbance of the experimental group-absorbance of DMSO.

### In vitro cell migration and invasion assay

At 24 h after transfection, the cells in different groups were treated with trypsin and re-suspended as single-cell solutions. A total of 2 × 10^5 ^cells in 0.5 ml of serum-free RPMI 1640 medium were seeded on a 8 μm-pore polycarbonate membrane Boyden chambers insert in a transwell apparatus (Costar, Cambridge, MA), either coated with (invasion) or without (migration) Matrigel (BD Biosciences, San Jose, CA). 600 μl RPMI1640 containing 20% FBS was added to the lower chamber. After the cells were incubated for 72 h (invasion) or 36 h (migration) at 37°C in a 5% CO_2 _incubator, the cells on the top surface of the insert were removed by wiping with a cotton swab. The cells that migrated to the bottom surface of the insert were fixed in 100% methanol for 2 min, stained in Giemsa for 2 min, rinsed in PBS and then subjected to microscopic inspection (×200). Values for invasion and migration were obtained by counting five fields per membrane and represented the average of three independent experiments [12].

### Statistics analysis

The data were presented as means-standard errors (SE) for MDA-MB-231 cells in each group. Statistical analysis was carried out by one-way ANOVA followed by Dunnett t-test or Student t-test (two means comparison). Statistical analysis was given using the related programs in SPSS 12.0. Differences were considered significant when P < 0.05.

## Results

### JMJD2A siRNA synthesis

The sequence of chemically synthesized JMJD2A siRNA was consistent with the requirements, and the purity reached to 98%. This met the experiment requirements.

### Observation of cell transfection results

**Figure 1:**
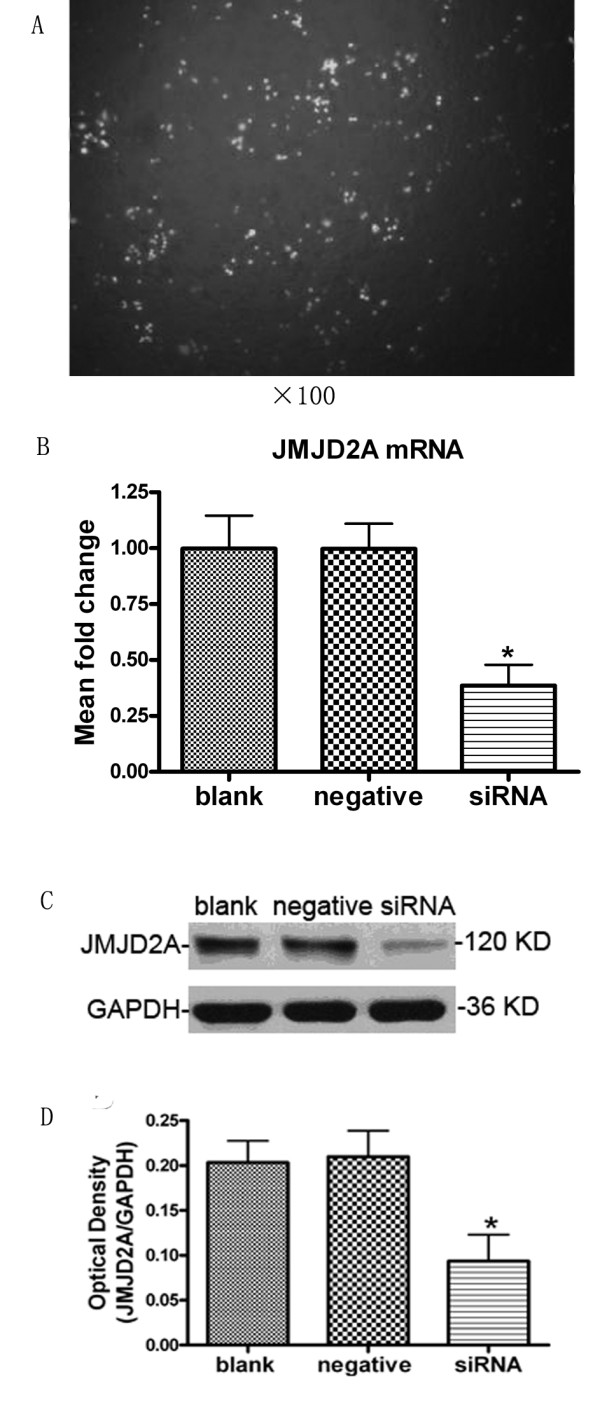
**Transfection was successful and levels of JMJD2A mRNA and protein were both down-regulated**. A. The green fluorescence cells transfected with FAM-siRNA under fluorescence microscope (Note: ×100). B. Column diagram analysis for mRNA levels of JMJD2A. JMJD2A-specific siRNA resulted in the reduction of JMJD2A mRNA levels in MDA-MB-231 cells. C. Western blot analysis for JMJD2A protein. D. Column diagram analysis for optical density by Western blotting. JMJD2A protein levels were down-regulated in siRNA group. (*P < 0.05, compared with blank control group and negative control group respectively)

MDA-MB-231 cells transfected with FAM-siRNA were subjected to Fluorescence microscopy at 8 h after transfection. The green fluorescence cells were considered to be transfected successfully. As shown in Figure 1A, cell transfection was successful and HiPerFect Transfection Reagent was effective. The transfection efficiency was about 72.3%.

### Transfection with JMJD2A-specific siRNA down-regulated JMJD2A mRNA levels to silence JMJD2A gene

According to the results of quantitative real-time PCR (Figure 1B), no significant difference (P > 0.05) was detected in the levels of JMJD2A mRNA between blank control group (0.998 ± 0.170) and negative control group (0.997 ± 0.150). The mRNA expression of siRNA group (0.386 ± 0.108) were significantly lower than that in blank control group (P < 0.05) and negative control group (P < 0.05), respectively. These data suggested that JMJD2A mRNA levels in MDA-MB-231 cells decreased significantly after transfection with JMJD2A siRNA. Transfection with JMJD2A-specific siRNA could result in JMJD2A mRNA degradation to silence JMJD2A gene.

### Transfection with JMJD2A-specific siRNA inhibited JMJD2A protein expression in MDA-MB-231 cells

Western blot analysis showed that, the levels of JMJD2A protein expression in the siRNA group (0.093 ± 0.051) were significantly lower than that in blank control group (0.203 ± 0.042) and negative control group (0.210 ± 0.050), respectively (P < 0.05; Figure 1C and 1D), while the difference between blank control group and negative control group was not significant (P > 0.05; Figure 1C and 1D). These data indicated that JMJD2A-specific siRNA silencing mRNA could significantly reduce the levels of JMJD2A protein expression in MDA-MB-231 cells.

### Silencing JMJD2A gene resulted in cell cycle changes and proliferation inhibition in MDA-MB-231 cells

**Figure 2:**
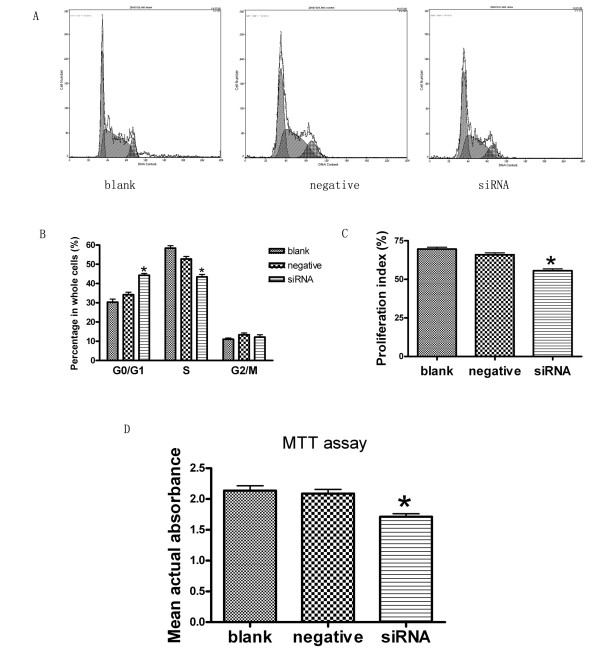
**Knock down of JMJD2A resulted in cell cycle change and proliferation inhibition**. A. DNA contents of MDA-MB-231 cells treated in blank control group, negative control group and siRNA group by FCM. B. Column diagram analysis for the percentages of cells at each phase in three different groups: G0/G1 phase, S phase and G2/M phase. At G0/G1 phase, there was a significant difference in the percentage of cells in siRNA group compared with blank control group and negative control group respectively. At S phase, there was a significant difference in the percentage of cells in siRNA group compared with blank control group and negative control group respectively, while no significant differences in the percentages of cells at G2/M phase in the three groups. C. Column diagram analysis for the proliferation indexes (PI) calculated in three different groups. PI in siRNA group was significantly lower than that in blank control group and negative control group respectively. D. Column diagram analysis for the actual absorbance of three different groups, the mean actual absorbance of siRNA group was significantly lower than that of the blank control group and the negative control group, respectively. (*P < 0.05, compared with blank control group and negative control group respectively)

Cell cycle analysis by FCM revealed that JMJD2A siRNA could induce changes in cell cycle of MDA-MB-231 cells. The mean value of the experiments was shown in Figure 2A, B and 2C. There were no significant differences (P > 0.05) in the percentages of cells at each phase between blank control group and negative control group. Compared with blank control group (30.3 ± 2.7%) and negative control group (34.2 ± 2.3%) respectively, there was a significant difference (P < 0.05) in the percentage of cells in G0/G1 phase in siRNA group (44.3 ± 1.6%). Similarly, there was a significant difference (P < 0.05) in the percentage of cells in S phase in siRNA group (43.4 ± 2.3%), versus blank control group (58.4 ± 2.1%) and negative control group (52.8 ± 2.2%), respectively. However, there was no significant difference (P > 0.05) in the percentage of cells in G2/M phase in siRNA group (12.1 ± 2.2%), relative to blank control group (11.0 ± 1.2%) and negative control group (13.3 ± 1.8%), respectively. Silencing JMJD2A gene could increase the percentage of cells at G0/G1 phase and decrease the percentage of cells at S phase. The results suggested that the treatment could arrest cells at the G1/S checkpoint and delay cell cycle into S phase. Furthermore, proliferation indexes (PI) of each group were calculated. We found that there was a significant difference (P < 0.05) in PI of siRNA group (55.6 ± 2.1%), versus blank control group (69.6 ± 2.1%) and negative control group (65.9 ± 2.2%), respectively. Our results revealed a change in cell cycle with transfection and indicated that cell proliferation could be inhibited by transfection.

Additionally, MTT assay was performed to test the effects of transfection with JMJD2A siRNA on the proliferation of MDA-MB-231 cells treated in three different groups. As shown in Figure 2D, there was no significant difference (P > 0.05) in the average actual absorbance between blank control group (2.136 ± 0.135) and negative control group (2.089 ± 0.115). The average actual absorbance in siRNA group (1.711 ± 0.087) was significantly lower than that in blank control group (P < 0.05) and negative control group (P < 0.05), respectively. Absorbance represents cell proliferation in MTT assay. The MTT assay results consistented with FCM results. These data indicated that transfection with JMJD2A siRNA could significantly reduce the proliferation of MDA-MB-231 cells.

### Silencing JMJD2A gene suppressed MDA-MB-231 cell migration and invasion in vitro

**Figure 3:**
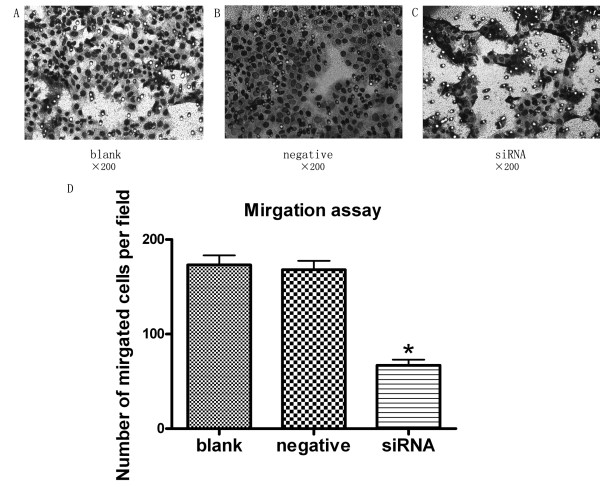
**Knock down of JMJD2A resulted in suppressing tumor cell migration**. A. Cells in blank control group transversed the Transwell membrane. B. Cells in negative control group. C. Cells in siRNA group. D. Column diagram analysis for the number of MDA-MB-231 cells in migration assay. The number of siRNA group (67 ± 10.2) was decreased compared with that of blank control group (173 ± 17.7) and negative control group (168 ± 16.4), respectively. (*P < 0.05, compared with blank control group and negative control group respectively) (Note: ×200)

**Figure 4:**
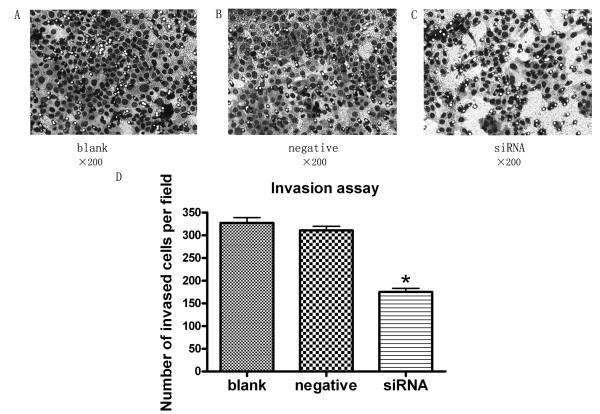
**Knock down of JMJD2A resulted in suppressing tumor cell invasion**. A. Cells in blank control group transversed the Transwell membrane. B. Cells in negative control group. C. Cells in siRNA group. D. Column diagram analysis for the number of MDA-MB-231 cells in invasion assay. The number of siRNA group (175 ± 14.4) was decreased compared with that of blank control group (327 ± 20.8) and negative control group (311 ± 15.3), respectively. (*P < 0.05, compared with blank control group and negative control group respectively) (Note: ×200)

As displayed in Figure 3, cell migration was significantly decreased in siRNA group than in blank control group (P < 0.05) and negative control group (P < 0.05), respectively. Cells in siRNA group showed significantly decreased invasiveness, compared with blank control group (Figure 4; P < 0.05) and negative control group (Figure 4; P < 0.05). These results demonstrated that transfection with JMJD2A siRNA could reduce the migration and invasion of MDA-MB-231 cells.

## Discussion

As leading cause of cancer death among females, human breast cancer has the features of powerful invasive ability and early metastatic property. Human breast cancer with the incidence rate increasing is the threat to human health. It is significantly meaningful to understand the pathologic mechanism of breast cancer and find treatment target site. Recent researches indicate that not only gene dysfunction but also histone modifications are involved in breast tumorigenesis [13].

Recent studies have implicated H3K9 modifications in numerous biological phenomena including germ cell development, × chromosome inactivation, DNA damage repair and apoptosis [14]. Recent reports also link deregulated histone methylation to tumorigenesis [15,16]. An H3K9 histone methyltransferase, Suv39H1, has been shown to function as a tumor suppressor by maintaining H3K9 methylation levels [17,18]. These data imply that H3K9me3 demethylases JMJD2A protein may take part in tumorigenesis through demethylation of H3K9me3.

Here we hypothesized that down-regulation of JMJD2A expression in MDA-MB-231 cell line would affect breast tumorigenesis and tumor biological characteristics. To test this hypothesis, JMJD2A-specific siRNA was transfected into human breast cancer cell line MDA-MB-231 to observe the effects. It was proved that JMJD2A gene could be silenced efficiently in MDA-MB-231 cell line by transfection with JMJD2A-specific siRNA and HiPerFect Transfection Reagent in this study. According to the results of Quantitative real-time PCR and Western blot analysis, the levels of JMJD2A mRNA and protein expression were both down-regulated based on the transfection. Further, FCM and MTT assay results showed cell cycle changes and proliferation inhibition existed in MDA-MB-231 cell line, and migration and invasion in vitro were both suppressed. These data imply tumor growth and metastasis may be restrained by silencing JMJD2A, and JMJD2A may be associated with breast cancer cell line MDA-MB-231, thus JMJD2A might be the potential therapeutic target in breast cancer.

However, the mechanism of JMJD2A in breast cancer is not very clear, here we discuss the probable role of JMJD2A in breast cancer based on our own recent data and the literature. Local chromatin architecture which is strongly influenced by post-translational modifications of histones like methylation is now generally recognized as an important factor in the regulation of gene expression [19,20]. The combination of different modifications and the incorporation of different histone variants which have distinct roles in gene regulation, have led to the proposition of a regulatory histone code which determines, at least partly, the transcriptional potential for a specific gene or a genomic region [21]. High endogenous expression of JMJD2A protein catalyzes demethylation of H3K9me3 excessively to break the balance between methylated and demethylated histones. Genome-wide studies show that H3K9me3 is enriched in heterochromatin, especially, as the mark with general repressive nature, H3K9me3 is predominant in coding regions of some active genes [22-25]. The intragenic permissive chromatin regions are flanked by the repressive mark, H3K9me3, and the maintenance of the intragenic chromatin boundary appears to functions as a checkpoint in elongation [26]. These data predict that the H3K9me3 demethylase activities of JMJD2A protein may act as transcriptional activators.

A recent research focusing on another member of JMJD2 family proteins JMJD2B, which is considered to have the similar function as JMJD2A in breast cancer demonstrated that JMJD2B constitutes a key component of the estrogen signaling pathway and the establishment of local epigenetic state and chromatin structure required for proper induction of ER responsive genes. JMJD2B which interacts with ERα and components of the SWI/SNF-B chromatin remodeling complex was recruited to ERα target sites, demethylated H3K9me3 and facilitated transcription of ER responsive oncogenes including MYB, MYC and CCND1, and knockdown of JMJD2B severely impaired estrogen induced cell proliferation and the tumor formation capacity of breast cancer cells as a consequence [27]. Consisting with that research, our data showed that silencing of JMJD2A could suppress the proliferation, migration and invasion of MDA-MB-231 cell line, thereby indicating that JMJD2A may be involved in the estrogen signaling pathway.

Though JMJD2A and 2B exhibited robust interactions with ER, in contrast to depletion of JMJD2B, depletion of JMJD2A caused only a marginal defect in ER target gene induction [27]. There may be another pathway JMJD2A involved in human breast cancer. It was described that JMJD2A has molecular characterization in binding both retinoblastoma protein (pRb) and histone deacetylases (HDACs) [28]. JMJD2A maybe associated with pRb recruits HDACs to the pRB-E2F complex, changes the chromatin structure at the E2F-responsive promoter and induced suppression of target gene E2F expression [29,30]. E2F1, 4 and their complexes with HDAC play an important role in downregulating the expression of the maternally imprinted tumor suppressor gene ARHI in breast cancer cells. Expression of ARHI is markedly down-regulated in breast cancer, and reactivation of ARHI expression in breast cancer cells is associated with decreased H3K9me3 which is demethylated by JMJD2A [31,32].

Together, JMJD2A may be, at least in part, involved in human breast cancer by constituting a key component of the estrogen signaling pathway or binding pRb and HDACs to suppress E2F-induced ARHI expression. However, the exact mechanism of JMJD2A in human breast cancer still remains elusive. The role of JMJD2A may be diverse rather than single.

To date, this is the first report highlighting that the suppression of proliferation, invasion and migration in human breast cancer cell line MDA-MB-231, at least in part, results from silencing of JMJD2A. The present study sheds light on the novel role of JMJD2A in breast cancer. However, our results were based on a single cell line. Further researches to determine the differential expression of JMJD2A between normal and cancer breast tissue and the mechanism of JMJD2A in breast cancer are required.

## Competing interests

The authors declare that they have no competing interests.

## Authors' contributions

BX-L and MC-Z carried out experiments and drafted the manuscript. CL-L and P-Y participated in study design and helped to draft the manuscript. H-L, HM-X, HF-X, YW-S and AM-X participated in study design, performed experiments and ZQ-Z participated in study design and revised manuscript. All authors approved the final manuscript.
